# Prospective evaluation of risk factors for acute adverse reactions to iodinated contrast media in contrast-enhanced CT

**DOI:** 10.3389/fmed.2026.1870320

**Published:** 2026-10-07

**Authors:** Ruigang Xie, Hui Zhang, Junjie Sun, Haibo Hu, U. Joseph Schoepf, Yinghui Ge

**Affiliations:** 1 Department of Radiology, Fuwai Central China Cardiovascular Hospital, Central China Fuwai Hospital of Zhengzhou University, Zhengzhou, Henan, China; 2 Department of Cardiac Surgery, Fuwai Central China Cardiovascular Hospital, Henan Provincial Clinical Research Center for Cardiovascular Disease, Zhengzhou, Henan, China; 3 Center of Structural Heart Disease, Fuwai Hospital, Chinese Academy of Medical Sciences, National Center for Cardiovascular Diseases, Beijing, China; 4 Department of Radiology and Radiological Science, Medical University of South Carolina, Charleston, SC, United States

**Keywords:** acute adverse reactions, allergic-like reactions, IgE, iodinated contrast media, physiologic reactions, risk factor

## Abstract

**Objective:**

This study aimed to identify clinical risk factors for acute adverse reactions (AARs) to iodinated contrast media (ICM) during contrast-enhanced CT.

**Methods:**

In this prospective study, A total of 1,462 patients with contrast-enhanced CT scans were continuously enrolled between October and December 2020 in a Class A tertiary cardiovascular specialty hospital. Blood samples were collected before the scan to determine serum immunoglobulin E (IgE), eosinophil (EOS) counts, and glucose levels. Demographic data and clinical characteristics were extracted from the hospital information system and pre-designed case report forms. Multivariate Firth logistic regression and receiver operating characteristic (ROC) analyses for these factors were performed to assess predictors of AARs.

**Results:**

Among 1,462 patients, 62 (4.24%) experienced AARs, including 20 (1.37%) acute allergic-like reactions. Cardiac insufficiency (OR = 1.983, 95% CI: 1.115–3.525), age (OR = 0.980, 95% CI: 0.961–0.999), ICM type (OR = 2.221, 95% CI: 1.320–3.737), CT examination type (OR = 0.492, 95% CI: 0.289–0.837), prior ICM reaction history (OR = 35.411, 95% CI: 6.408–195.700), and allergy history (OR = 3.327, 95% CI: 1.655–6.688) were independent risk factors for AARs. Hyperlipidemia might be potentially associated with AARs. Elevated baseline IgE, ICM type, and gout might be potentially associated with acute allergic-like reactions. EOS and blood glucose levels showed no significant association.

**Conclusion:**

Cardiac insufficiency, age, ICM type, CT examination type, prior ICM reactions, and allergy history are key risk factors for AARs. The following exploratory associations require multi-center validation: hyperlipidemia may have a potential association with AARs; baseline IgE levels, ICM type and gout may be associated with acute allergic-like reactions.

## Highlights

- Cardiac insufficiency, age, iodinated contrast media type, CT examination type, previous history of adverse reactions to iodinated contrast media, and a history of various allergies increased the odds of acute adverse reactions to iodinated contras media.
- Hyperlipidemia was a potential predictor of acute adverse reactions to iodinated contrast media.
- IgE level might be a potential predictor of acute allergic-like reactions to iodinated contrast media.

## 1 Introduction

Iodinated contrast media (ICM) are widely employed in clinical practice to enhance the diagnostic efficacy of medical imaging. However, the increasing utilization of these agents has been accompanied by a rising incidence of adverse reactions (1, 2). The adverse reactions to ICM can be classified into acute or delayed. Acute adverse reactions (AARs) can further be categorized as either allergic-like or physiologic, and organized into three general categories of severity (mild, moderate, or severe). AARs typically occur within 1 h after ICM administration, mostly mild, such as rash, itching, nausea and vomiting, but occasionally serious, leading to anaphylactic shock or death. Nearly all life-threatening reactions occur within 20 min, with some resulting in death (1 fatality per 170,000 low-osmolality ICM administrations) (3–5). In recent years, there has been a reported increase in the incidence and severity of AARs to ICM. However, the conclusions regarding these trends remain inconclusive. Previous studies have predominantly relied on retrospective data, largely attributed to the infrequent and unpredictable nature of AARs. However, retrospective investigations may overlook certain contributing factors to these reactions, and findings regarding the risk factors for AARs may differ between prospective and retrospective study designs (5–7).

Physiologic reactions are primarily associated with the physicochemical properties of ICM. In contrast, allergic-like reactions — often termed hypersensitivity reactions — are linked to individual patient susceptibility. These reactions are also referred to as anaphylactoid reactions, IgE-mediated or non-IgE-mediated allergic reactions, and non-allergic hypersensitivity reactions induced by ICM (8, 9). Previous studies have confirmed that allergic-like reactions to ICM, especially acute allergic reactions, are mediated by IgE (10). Some authors have studied the correlation and diagnostic value of baseline total IgE and eosinophils (EOS) to allergic diseases such as asthma (11–14). While the correlation between total IgE levels, peripheral blood EOS levels and acute allergic-like reactions to ICM is still unclear. Furthermore, in routine clinical practice, patients are typically required to fast (15) for 4–6 h or longer prior to undergoing contrast-enhanced CT examinations. During this fasting period, patients’ blood glucose levels can fluctuate. Therefore, it is crucial to further investigate whether blood glucose levels are associated with acute allergic-like reactions to ICM. This prospective study aims to identify clinical and biochemical predictors of AARs to ICM. Our findings are intended to assist in the proactive identification of high-risk individuals, enabling clinicians to optimize the safety profile of contrast-enhanced examinations.

## 2 Methods

### 2.1 Study participants

This prospective study was performed in accordance with the Declaration of Helsinki and received institutional review board approval (Central China Fuwai Hospital, ethics number: 2021 Lunshen No. 2), all participants gave written informed consent prior to examination.

Patients who underwent contrast-enhanced CT examinations in a Class A tertiary cardiovascular specialty hospital between October and December 2020 were enrolled continuously in this study. This study was registered in the Chinese Clinical Trial Registry (registration number: ChiCTR2000040275, 27/11/2020).

The inclusion criteria were (a) patients referred to undergo contrast-enhanced CT examination, and (b) patients who agreed to sign the “Informed Consent for Contrast-Enhanced CT Examination” form.

The exclusion criteria included (a) patients aged ≤18 years, (b) pregnant or lactating patients, (c) patients with a history of severe adverse reactions to ICM, (d) patients with severe renal dysfunction (creatinine clearance rate <30 mL/min), (e) patients who previously participated in a similar study, (f) patients perceived to be unwilling to complete the questionnaire (Supplementary Material Attachment 1) or follow-up via telephone, and (g) patients with uncontrolled hyperthyroidism.

Of note, no patients included in this study received any prophylactic premedication, such as systemic corticosteroids or antihistamines, prior to the contrast-enhanced CT examination.

Sample size calculation was performed using the Riley method. The results indicated that a minimum sample size of 1,397 subjects is required for the study on AARs to ICM, with at least 60 positive outcome events. The specific methods and procedures for parameter estimation are detailed in Supplementary Appendix 1.

### 2.2 CT examination scheme and blood sample testing

A third-generation dual-source CT (Somatom Force, Siemens Healthineers, Forchheim, Germany) was used in this clinical trial. Nonionic ICM was intravenously injected using a high-pressure injection system. There are two types of ICM used, iopromide 370 (Bayer Healthcare, Leverkusen, Germany) and iodixanol 320 (Jiangsu Hengrui Medicine Co., Ltd., Jiangsu, China). The selection between iopromide and iodixanol was performed randomly. The ICM was extrinsically warmed to 36℃–37℃ in an external incubator before use.

Fingertip blood drop (for immediate blood glucose) and venous blood sample (3 mL, for IgE and/or EOS) were collected before contrast-enhanced CT procedure. A blood glucose test strip and blood glucose meter (Accu-Chek Active, Roche Diabetes Care GmbH, Mannheim, Germany) were used for blood glucose testing. Serum IgE was detected via electrochemiluminescence using an IgE detection kit (Elecsys IgE Ⅱ, Roche Diagnostics GmbH, Mannheim, Germany). The detection system used was Roche cobas 8000 E602 (Cobas, Roche Diagnostics GmbH, Mannheim, Germany).

EOS counts were determined via flow cytometry using a semiconductor laser-based cytometric method (BC-6900 Auto Hematology Analyzer, Shenzhen Mindray Bio-medical Electronics Co. Ltd., Shenzhen, China).

IgE precision determination is as follow. (a) Experimental protocol: Performance verification was conducted using two levels of control samples (high and low concentrations, aligned as closely as possible with medical decision limits). The samples were analyzed daily in triplicate over a 5-day period. All raw data were recorded and subsequently processed using standardized performance verification software to automatically calculate the within-run imprecision, intermediate (between-run) imprecision, and total imprecision. (b) Performance requirements for the detection system: The allowable coefficient of variation (CV) was determined based on the general standard. For analytes lacking specific industry standards, the allowable total error (TEa) from proficiency testing (PT) or external quality assessment (EQA) programs was adopted; otherwise, quality specifications derived from biological variation or manufacturer's claims were utilized. The analytical performance goals were defined as: within-run imprecision <1/4 TEa and intermediate imprecision <1/3 TEa. For the IgE assay, the TEa is 25%, establishing a requirement of ≤6.25% for within-run imprecision and ≤8.33% for intermediate precision (measured in IU/mL) (Supplementary Material Attachment 2).

Regarding the precision of IgE: its total mean (unit:IU/mL), within-run standard deviation (SWR, representing repeatability), coefficient of variation of SWR(CVWR), laboratory-wide intermediate precision (SWL, representing intermediate precision), and coefficient of variation of SWL(CVWL) are as follows: Level 1: 121.667, 2.3944, 1.97%, 3.8283, 3.15%; Level 2: 295.467, 2.0166, 0.68%, 2.7325, 0.92%. Both Level 1 and Level 2 precision tests meet the required criteria (Supplementary Material Attachment 2).

The mean (unit:IU/mL), standard deviation(SD), and coefficient of variation (CV) for IgE calibration are as follows: Level 1: 124.973, 4.811, 3.849; Level 2: 323.918, 11.299, 3.488. Both Level 1 and Level 2 quality control measures comply with the required standards (Supplementary Material Attachment 3).

The precision and reproducibility of the EOS assay met routine requirements (Supplementary Material Attachment 4). For EOS quality control, there are three levels of calibration materials: The mean value (unit:%), standard deviation(SD), and coefficient of variation (CV) for EOS calibration are as follows: Level 1: 14.152, 2.962, 20.931; Level 2: 10.235, 0.519, 5.068; Level 3: 8.653, 2.665, 30.792. All Level 1, Level 2, and Level 3 quality control measures comply with the required standards (Supplementary Material Attachment 5).

More details regarding laboratory methods and assay reproducibility — details on inter-assay variability, precision, and laboratory quality control are provided in the Supplementary Material (Attachment 2,3,4,5).

### 2.3 Study procedures and data analysis

The clinical trial process is illustrated in Figure 1. Among the 1,462 patients, 241 did not consent to blood sample collection; 626 agreed to blood sample collection and measurement of blood glucose, IgE, and EOS levels; 770 agreed to the collection of blood samples and measurement of baseline IgE and EOS levels; and 1,077 agreed to the measurement of immediate blood glucose levels.

**Figure 1:**
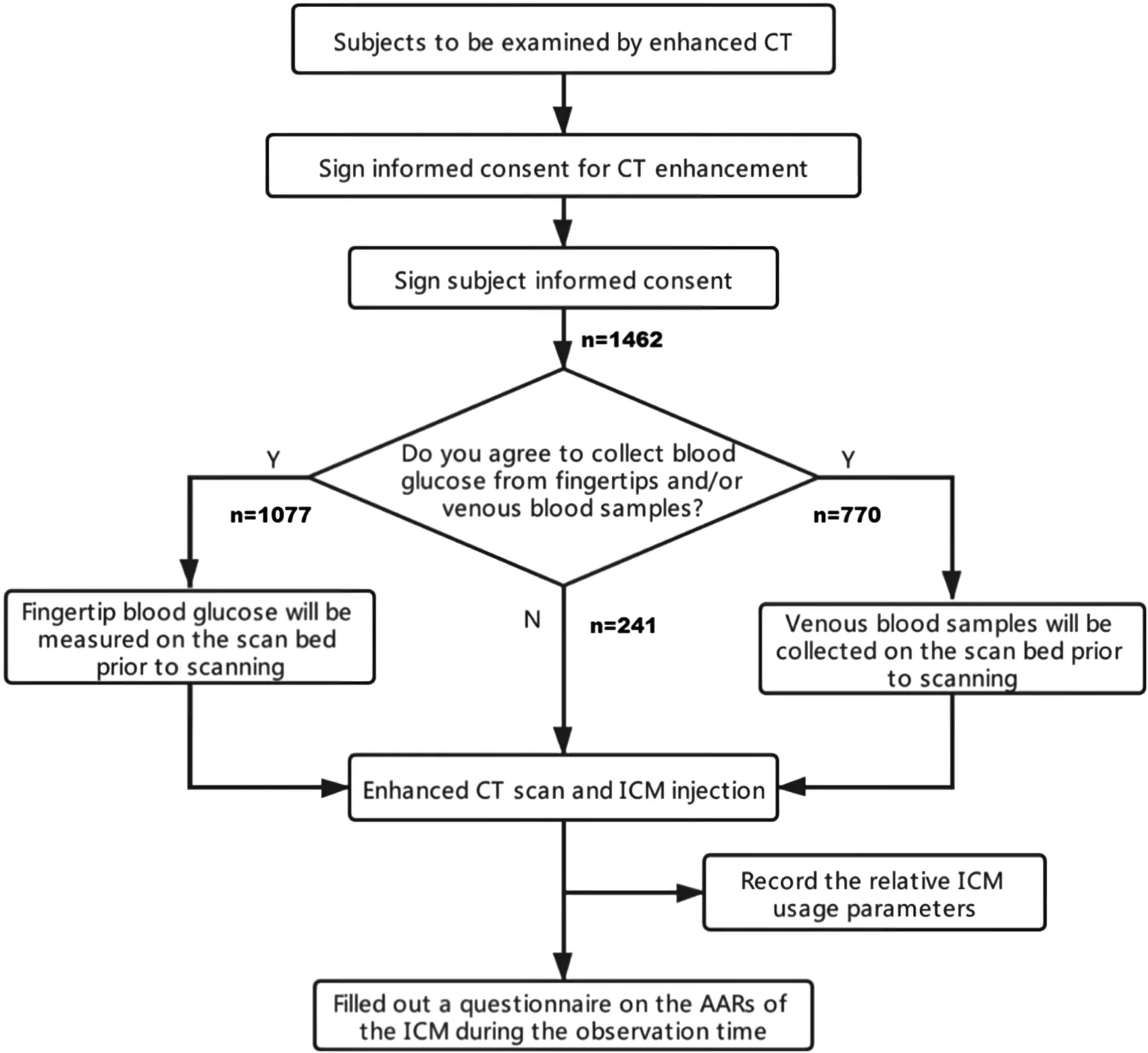
Clinical trial flow chart. A total of 1,462 patients were initially enrolled in the study. Of these, 241 patients did not consent to blood sample collection, while 626 patients agreed to blood sample collection and measurement of blood glucose, IgE, and EOS levels. Additionally, 770 patients consented to blood sample collection and measurement of baseline IgE and EOS levels, and 1,077 patients agreed to measurement of immediate blood glucose levels. The figure shows the distribution of patients across different stages of the study, including enrollment, blood sample collection, and measurement of various biomarkers. CT, computed tomography; ICM, iodinated contrast media; AARs, acute adverse reactions; IgE, immunoglobulin E; EOS, eosinophil.

Demographic and clinical characteristics were retrieved from the hospital information system and our self-designed case report forms. The collected variables included age, sex, height, weight, body mass index (BMI), systolic blood pressure (SBP), diastolic blood pressure (DBP), and a history of comorbidities such as hypertension, coronary heart disease (CHD), hyperlipidemia, cardiac insufficiency, diabetes mellitus (DM), chronic kidney disease (CKD), thyroid disease, and gout. Allergic history — defined as a prior history of adverse reactions to ICM or other allergic conditions — was also recorded. All diagnostic information was extracted from the electronic medical records. Allergic diseases were defined as the presence of one or more of the following conditions: bronchial asthma, allergic rhinitis, atopic dermatitis, and urticaria. A history of allergies was defined as the presence of one or more of the following: drug allergies [including antibiotics, non-steroidal anti-inflammatory drugs (NSAIDs), sulfa, or purine-related drugs] and food allergies.

The type of CT examination [coronary CT angiography (CCTA) vs. non-CCTA], the type of ICM used (iodixanol or iopromide), injection volume, and flow rate were all recorded. Serum creatinine levels were retrieved from the patients’ electronic medical records.

Furthermore, baseline levels of IgE, EOS, and point-of-care blood glucose measured in the CT suite prior to the scan were also documented.

The occurrence, classification, and grading of AARs to ICM were determined in accordance with the 2020 American College of Radiology Manual on Contrast Media (16). Patients were categorized into two main groups: the AARs group and the non-reaction group. The AARs group was further stratified into allergic-like and physiologic reactions subgroups. Per the guidelines, mild AARs are self-limited and show no evidence of progression; moderate AARs are more pronounced and typically necessitate medical intervention; and severe AARs are potentially life-threatening, often requiring intensive care or carrying the risk of permanent morbidity or mortality. The final assessment of AARs was conducted independently by two blinded clinicians, with any discrepancies resolved through consultation with a third senior physician.

### 2.4 Statistical analysis

Statistical analysis was performed using R software (version 4.5.3). The categorical variables were expressed as frequency and composition ratio (%), and the continuous variables conforming to a skew distribution were expressed as median and upper and lower quartile *M* (*P*25, *P*75). Continuous variables with non-normal distribution were compared using Mann–Whitney U test, and the categorical variables were compared using chi-squared test. Firth logistic regression was employed to evaluate the risk factors. First, we conducted univariate Firth logistic regression; then, the variables with *P* < 0.1 in the univariate Firth logistic regression analysis were included as candidate variables in the multivariate Firth logistic regression analysis. A Firth logistic regression model was used to analyze the independent predictors of AARs and acute allergic-like reactions, and the receiver operating characteristic (ROC) curves in patients with acute allergic-like reactions was depicted. Internal validation was performed using the bootstrapping method. Model calibration was investigated using calibration curves, and model goodness-of-fit was assessed with the Hosmer-Lemeshow test. All *P*-values were two-sided, and a *P*-value < 0.05 was considered statistically significant.

## 3 Results

### 3.1 Comparison of clinical baseline data of patients with and without AARs

This study included 1,462 patients, of whom 62/1462 (4.24%) experienced AARs to ICM. The patients’ age and baseline creatinine value in AARs positive group were lower than those in negative group. The higher injection velocity of ICM, CCTA examination type, hyperlipidemia, history of adverse reactions to ICM and history of various allergies in AARs positive group were statistically significant than those in negative group. Their clinical characteristics are summarized in Table 1.

**Table 1:** Demographic and clinical characteristics of patients undergoing contrast-enhanced CT.

Characteristic	All (1,462)	AARs (-) (1,400)	AARs (+) (62)	*P*
Age(y)	60 (51,68)	60 (51,69)	56 (48,64)	0.049
Sex				0.216
Male	911 (62.3%)	877 (62.6%)	34 (54.8%)	
Female	551 (37.7%)	523 (37.4%)	28 (45.2%)	
Height(cm)	165.0 (159.0,171.0)	165.5 (159.0,171.0)	164.0 (155.0,172.0)	0.277
Weight(kg)	70.0 (60.0,78.0)	70.0 (60.0,78.0)	70.0 (62.0,77.5)	0.916
BMI(kg/m^2^)	25.5 (23.1,27.8)	25.4 (23.1,27.8)	26.3 (23.3,28.3)	0.407
SBP(mmHg)	132 (120,147)	132 (120,147)	132 (120,144)	0.419
DBP(mmHg)	84 (76,93)	84 (76,93)	84 (74,94)	0.405
ICM type				0.004
Iodixanol	872 (59.6%)	846 (60.4%)	26 (41.9%)	
Iopromide	590 (40.4%)	554 (39.6%)	36 (58.1%)	
ICM injection volume (mL)	60 (60,80)	65 (60,80)	60 (60,80)	0.698
ICM injection flow rate (mL/s)	4.5 (4.0,4.5)	4.5 (4.0,4.5)	4.5 (4.5,4.5)	0.040
CT examination type				0.005
CCTA	596 (40.8%)	560 (40.0%)	36 (58.1%)	
Non-CCTA	866 (59.2%)	840 (60.0%)	26 (41.9%)	
Hypertension	722 (49.4%)	695 (49.6%)	27 (43.5%)	0.315
CHD	636 (43.5%)	605 (43.2%)	31 (50.0%)	0.320
Hyperlipidemia	131 (9.0%)	116 (8.3%)	15 (24.2%)	<0.001
Cardiac insufficiency	339 (23.1%)	320 (22.9%)	19 (30.6%)	0.144
Diabetes mellitus	326 (22.3%)	314 (22.4%)	12 (19.4%)	0.547
Chronic kidney disease	59 (4.0%)	56 (4.0%)	3 (4.8%)	1.000
Thyroid disease	231 (15.8%)	225 (16.1%)	6 (9.7%)	0.169
Gout	9 (0.6%)	8 (0.6%)	1 (1.6%)	0.326
Allergic disease ^a^	43 (2.9%)	41 (2.9%)	2 (3.2%)	1.000
History of adverse reactions to ICM	5 (0.3%)	2 (0.14%)	3 (4.8%)	0.001
History of various allergies ^b^	106 (7.3%)	95 (6.8%)	11 (17.7%)	0.003
Baseline creatinine (*μ*mol/L)	69.0(58.0,80.0)	69.0(58.0,80.0)	64.0(53.0,75.0)	0.041

a Allergic disease is defined as having one or a combination of the following: bronchial asthma, allergic rhinitis, atopic dermatitis, and urticaria.

b History of various allergies is defined as having one or a combination of the following: drug allergy (antibiotics, NSAIDs, sulfa, or purine), food allergy. AARs acute adverse reactions; BMI, body mass index; SBP, systolic blood pressure; DBP, diastolic blood pressure; CHD, coronary heart disease; ICM, iodinated contrast media; CCTA, cardiac computed tomography angiography.

### 3.2 Clinical characteristics of patients with AARs: distribution by system involvement, age, and BMI

Of these 62 AARs, 54 (3.69%), 7 (0.48%), and 1 (0.07%) patient experienced mild, moderate, and severe reactions. This included 20/1462 (1.37%) cases of acute allergic-like reactions and 42/1462 (2.87%) cases of physiologic reactions, respectively.

The systematic distribution of the clinical manifestations of AARs to ICM was as follows: the incidence of nervous system symptoms, mainly dizziness, was the highest (41.9%), gastrointestinal symptoms followed, accounting for 30.6%, skin symptoms such as itching, rash and erythema reached 27.4%, cardiovascular symptoms reached 8.1%, and respiratory symptoms reached 3.2% (Figure 2).

**Figure 2:**
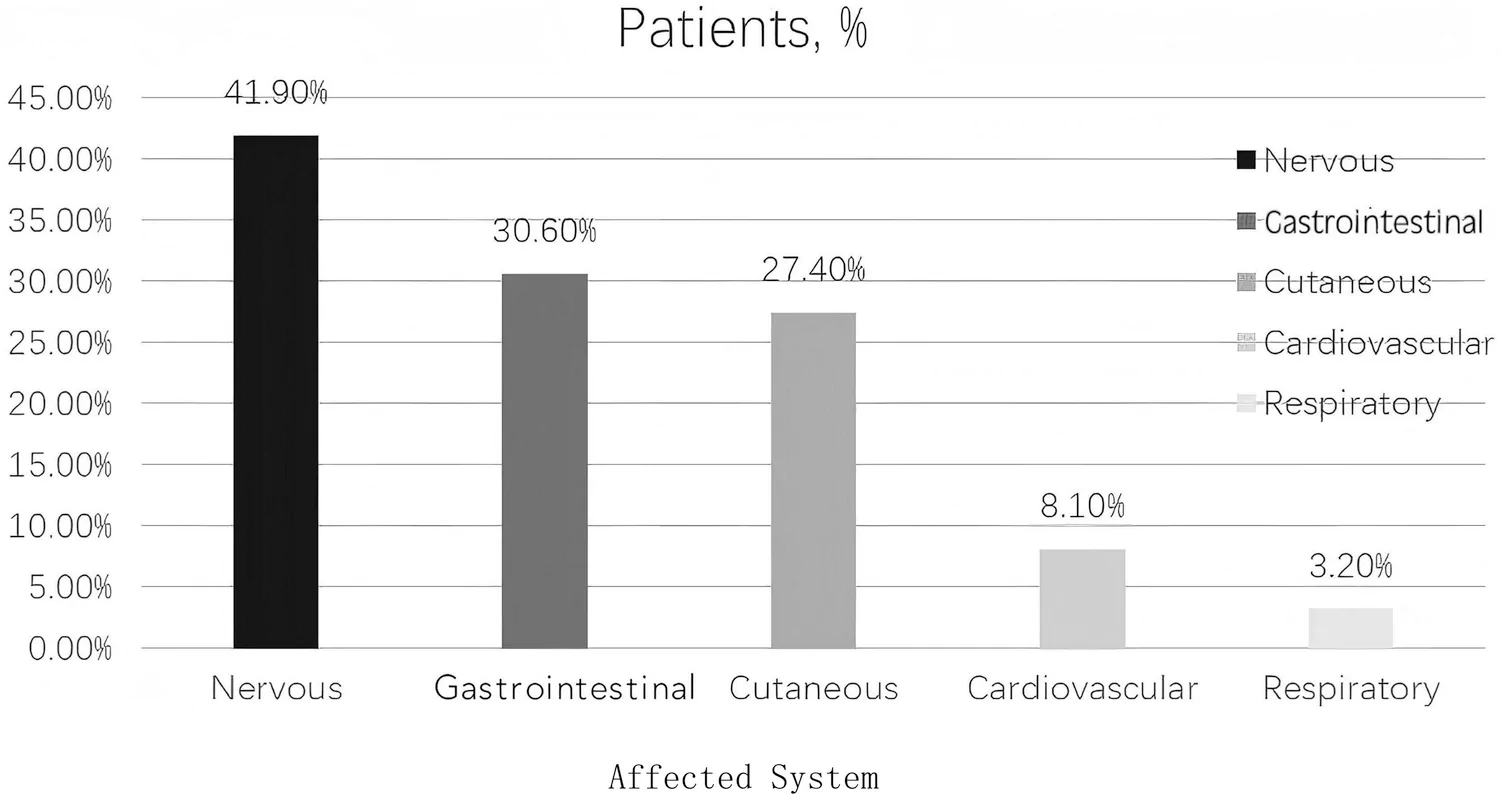
Systemic distribution of clinical manifestations in patients with AARs to ICM (*n* = 62). The most frequently involved system was the nervous system (41.9%, predominantly dizziness), followed by gastrointestinal symptoms (30.6%), cutaneous reactions (27.4%, including itching, rash, and erythema), cardiovascular symptoms (8.1%), and respiratory symptoms (3.2%). The data represent the proportion of patients experiencing each type of manifestation among the 62 cases of AARs recorded during contrast-enhanced CT examinations. AARs, acute adverse reactions; ICM, iodinated contrast media; CT, computed tomography.

The AARs patients were grouped by decade as follows: (−29), (30–39), (40–49), (50–59), (60–69), (70–79) and (80-) years group. The results showed that the incidence of acute allergic-like reactions was higher in patients aged 30–59 years, while the overall incidence of AARs was higher in patients aged 40–69 years, among which the highest incidence of AARs was 6.57% in patients aged 40–49 years. Comparisons of adverse event rates across different age groups were conducted using chi-square tests. Neither the incidence of AARs (*χ*2 = 8.876, *P* = 0.181) nor the incidence of allergic-like reactions (*χ*2 = 5.550, *P* = 0.475) showed statistically significant differences between age groups (Figure 3).

**Figure 3:**
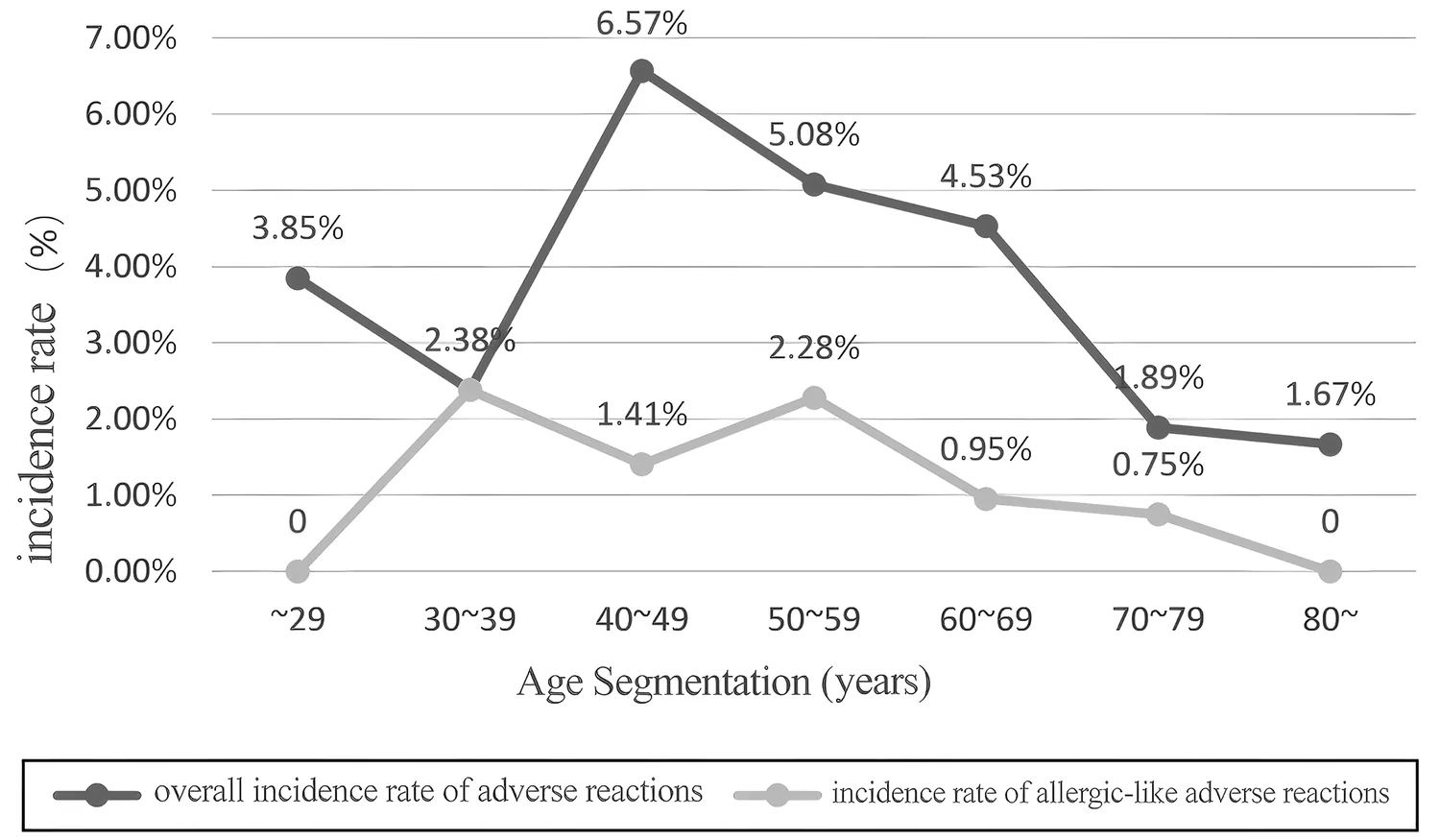
Age-specific incidence of overall AARs and acute allergic-like reactions among 1,462 patients. Patients were stratified by decade (from <30 to ≥80 years). The overall incidence of AARs (Chi-square tests: *χ*2 = 8.876, *P* = 0.181) was highest in the 40–49 years group (6.57%), while acute allergic-like reactions (Chi-square tests: *χ*2 = 5.550, *P* = 0.475) were more frequently observed in patients aged 30–59 years. AARs, acute adverse reactions.

The AARs patients were grouped by BMI as follows: [−18.5), [18.5–24), [24–28), [28–32) and [32-) kg/m^2^ group. Both the overall incidence of AARs and the incidence of acute allergic-like reactions were highest in the BMI segment [28–32) kg/m^2^. Chi-square tests indicated that there were no statistically significant differences in the incidence of AARs (*χ*2 = 8.106, *P* = 0.150) or allergic-like reactions (*χ*2 = 5.119, *P* = 0.402) among the different BMI groups (Figure 4).

**Figure 4:**
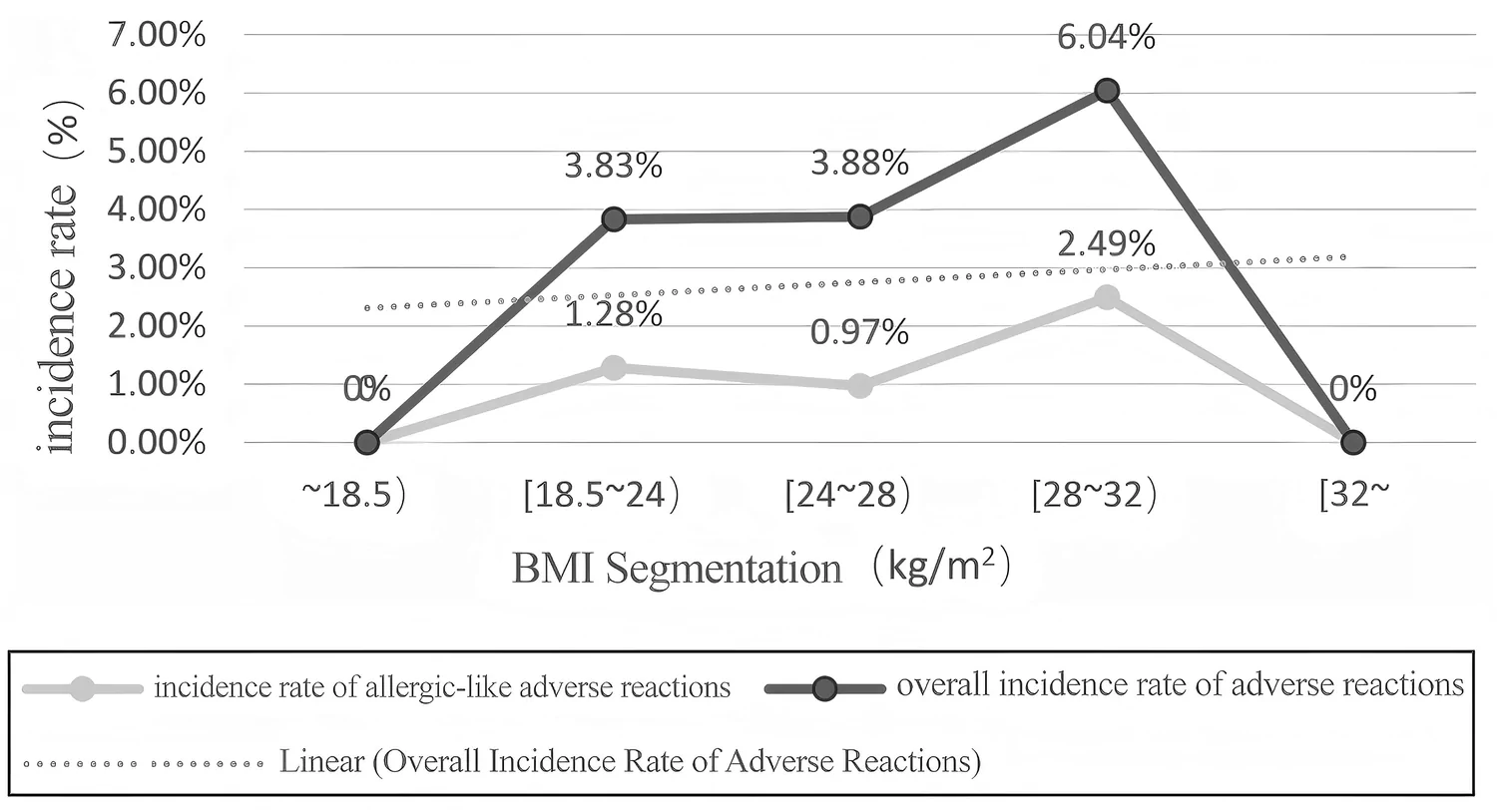
Incidence of overall AARs and acute allergic-like reactions across different BMI categories (*n* = 1,462). Patients were stratified into five BMI groups: <18.5, 18.5–24, 24–28, 28–32, and ≥32 kg/m^2^. The highest overall incidence of AARs (Chi-square tests: *χ*2 = 8.106, *P* = 0.150) and the highest incidence of acute allergic-like reactions (Chi-square tests: *χ*2 = 5.119, *P* = 0.402) were both observed in the 28–32 kg/m^2^ group. AARs, acute adverse reactions; BMI, body mass index.

### 3.3 Firth logistic regression model of AARs and clinical baseline data

#### 3.3.1 Univariate and multivariate firth logistic regression model of AARs and clinical baseline data of the patients when the blood samples were not considered

The inter-group differences in the clinical baseline data of the patients before ICM administration between the AARs and non-reaction groups were analyzed when the blood samples were not considered.

Univariate Firth logistic regression analysis revealed that high injection flow rate at 4.5 mL/s [odds ratio (OR) = 2.486, 95% confidence interval (CI): 1.071–5.771, *P* = 0.021], CCTA examination (vs. non-CCTA, OR = 0.484, 95% CI: 0.290–0.807, *P* = 0.005), hyperlipidemia (OR = 3.598, 95% CI: 1.967–6.581, *P* < 0.001), history of adverse reactions to ICM (OR = 32.906, 95% CI: 6.360–170.254, *P* *<* 0.001), history of various allergies (OR = 3.053, 95% CI: 1.559–5.978, *P* = 0.003), low baseline serum creatinine level (OR = 0.983, 95% CI: 0.968–0.998, *P* = 0.025), and type of ICM used with Iopromide (vs. Iodixanol, OR = 2.103, 95% CI: 1.261–3.507, *P* = 0.004) comprised the important risk factors for AARs to ICM. The results with a *P*-value of <0.1 in the univariate Firth logistic regression analysis were included as candidate variables in the multivariate Firth logistic regression alongside the possible clinical risk factors, such as sex, cardiac insufficiency, and thyroid disease. Furthermore, multivariate Firth logistic regression analysis revealed that hyperlipidemia, cardiac insufficiency, age, ICM type, CT examination type, history of adverse reactions to ICM, and history of various allergies were independent risk factors of AARs (Table 2).

**Table 2:** Multivariate firth logistic regression model of AARs.

Characteristic	AARs			
	Unadjusted OR (95% CI)	*P*	Adjusted OR (95% CI)	*P*
Hyperlipidemia (+)	3.598 (1.967–6.581)	<0.001	3.134 (1.689–5.816)	<0.001
Cardiac insufficiency (+)	1.511 (0.873–2.616)	0.150	1.983 (1.115–3.525)	0.026
Age (+)	0.984 (0.965–1.002)	0.091	0.980 (0.961–0.999)	0.048
ICM type (+)	2.103 (1.261–3.507)	0.004	2.221 (1.320–3.737)	0.028
CT examination type (+)	0.484 (0.290–0.807)	0.005	0.492 (0.289–0.837)	0.010
History of adverse reactions to ICM (+)	32.906 (6.360–170.254)	<0.001	35.411 (6.408–195.700)	<0.001
History of various allergies ^b^ (+)	3.053 (1.559–5.978)	0.003	3.327 (1.655–6.688)	0.002

b History of various allergies is defined as having one or a combination of the following: drug allergy (antibiotics, NSAIDs, sulfa, or purine), food allergy. AARs, acute adverse reactions; CI, confidence interval; OR, odds ratio; ICM, iodinated contrast media; CCTA, cardiac computed tomography angiography.

#### 3.3.2 Univariate and multivariate firth logistic regression model of acute allergic-like reactions and clinical baseline data of the patients when the blood samples were considered

##### 3.3.2.1 The intra-group differences in the blood samples of IgE, EOS, and immediate blood glucose levels of the patients before ICM administration between the allergic-like and non-allergic-like reactions groups were analyzed when the blood samples were considered

The differences in the baseline IgE (*Z* = −3.869, *P* *<* 0.001) and EOS (*Z* = −2.504, *P* *=* 0.012) levels were statistically significant in the allergic-like and non-allergic-like reactions groups, nor were the immediate blood glucose levels (*Z* = −0.298, *P* *=* 0.766) (Table 3).

**Table 3:** Analysis of the intragroup differences in IgE, EOS, and blood glucose levels.

Characteristic	Allergic-like reactions	Non-allergic-like reactions	*Z*	*P*
Baseline IgE levels	479.00 (164.85,759.05)	44.26 (17.08,110.95)	−3.869	<0.001
EOS levels	0.24 (0.18,0.34)	0.11 (0.07,0.19)	−2.504	0.012
Blood glucose levels	5.80 (5.50,7.40)	6.00 (5.40,5.00)	−0.298	0.766

IgE, immunoglobulin E; EOS, eosinophil.

##### 3.3.2.2 Analysis of the risk factors of acute allergic-like reactions to ICM

Of the patients who agreed to blood sample collection and measurement of IgE, nine patients experienced acute allergic-like reactions. Univariate Firth logistic regression analysis revealed that baseline IgE levels (OR = 1.00, 95% CI: 1.00–1.00, *P* = 0.002), the type of ICM used with Iopromide (vs. Iodixanol, OR = 4.34, 95% CI: 1.17–16.05, *P* = 0.024) and gout (OR = 29.71, 95% CI: 4.15–212.52, *P* = 0.010) were risk factors of acute allergic-like reactions. The baseline IgE levels, type of ICM used with Iopromide, and variables with a *P*-value of <0.1 in the univariate Firth logistic regression analysis, alongside the possible clinical risk factors, such as peripheral blood EOS levels were included in the multivariate Firth logistic regression analysis. Multivariate Firth logistic regression analysis revealed that baseline IgE levels (OR = 1.00, 95% CI: 1.00–1.00, *P* = 0.003), the type of ICM used with Iopromide (vs. Iodixanol, OR = 4.69, 95% CI: 1.24–17.68, *P* = 0.020) and gout (OR = 29.48, 95% CI: 2.98–291.82, *P* = 0.019) were independent predictors of acute allergic-like reactions to ICM (Table 4).

**Table 4:** Multivariate firth logistic regression model of acute allergic-like reactions for 770 patients with IgE blood samples.

Characteristic	*β*	OR	*P*	95% CI
Intercepts	−5.569	0.00	<0.001	(0.00–0.01)
Baseline IgE levels	0.002	1.00	0.003	(1.00–1.00)
ICM type	1.545	4.69	0.020	(1.24–17.68)
Gout	3.384	29.48	0.019	(2.98–291.82)

IgE, immunoglobulin E; ICM, iodinated contrast media.

##### 3.3.2.3 ROC curve of baseline IgE levels to predict the occurrence of acute allergic-like reactions to ICM

To further investigate the predictive effect of baseline IgE levels on the occurrence of acute allergic-like reactions, we depicted the ROC curve (Figure 5). The probability threshold was 0.012, the corresponding total IgE concentration was 158.1 IU/mL, and the area under the ROC curve was 0.868 (95% CI: 0.796–0.940, *P* = 0.003). At the optimal cut-off value, the sensitivity and specificity of baseline IgE levels in predicting acute allergic-like reactions were 88.9% and 86.6%, respectively.

**Figure 5:**
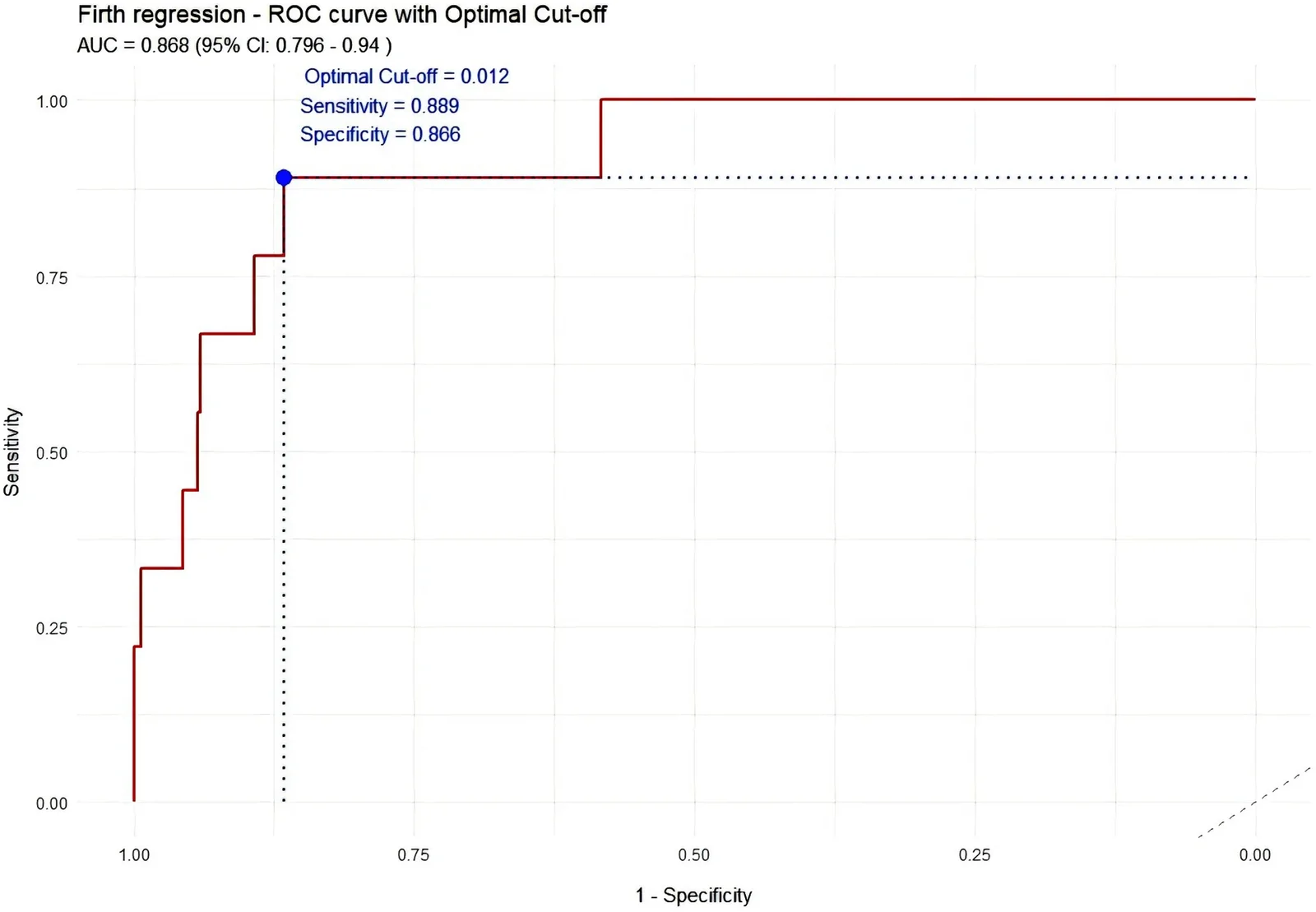
The ROC curve for baseline igE levels as a potential predictor of acute allergic-like reactions. The area under the ROC curve (AUC) was 0.868 (95% CI: 0.796–0.940, *P* = 0.003). The probability threshold is 0.012, and the corresponding optimal cut-off value for baseline IgE levels was determined to be 158.1 IU/mL, with a sensitivity of 88.9% and a specificity of 86.6%. This figure visually represents the exploratory finding of a potential association between elevated baseline IgE levels and the occurrence of acute allergic-like reactions to ICM. ROC, receiver operating characteristic; IgE, immunoglobulin E; AUC, area under the curve; CI, confidence interval; ICM, iodinated contrast media.

We further performed internal validation using the bootstrapping method. The results showed a 95% confidence interval (CI) for the AUC of (0.828, 0.972). To assess the model's calibration, we generated a calibration curve (Figure 6). The Hosmer-Lemeshow goodness-of-fit test indicated a good fit, with a *χ*2 value of 6.934 and a *P*-value of 0.544 (*P* > 0.05). This suggests that the predicted probabilities of the model align well with the observed outcomes, indicating good model calibration.

**Figure 6:**
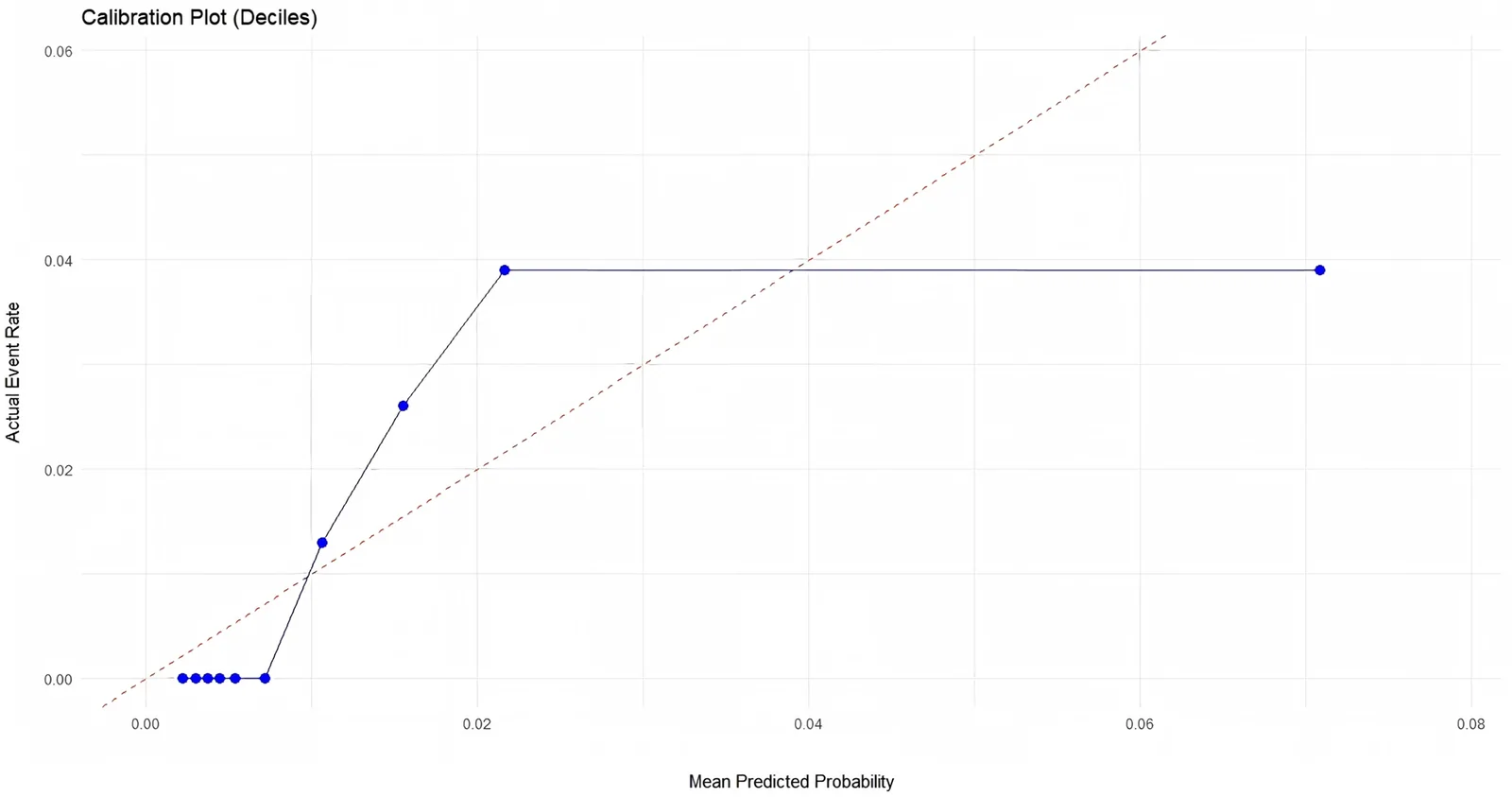
Calibration plots for 770 IgE blood samples. The Hosmer-Lemeshow goodness-of-fit test yielded the following results: *χ*2 = 6.934, *P* = 0.544. This indicates that there is no significant difference between the predicted probabilities of the model and the actual observed outcomes, suggesting that the model exhibits good calibration. IgE, immunoglobulin E

##### 3.3.2.4 Sensitivity analyses

Considering that the 95% CI for ‘history of previous contrast reaction’ was excessively wide in the initial model, a sensitivity analysis was performed by refitting the multivariate Firth regression model after excluding this variable to assess the stability of our findings. The results demonstrated that the odds ratios (ORs) and statistical significance of the other key risk factors remained largely consistent (Supplementary Appendix 4,5).

## 4 Discussion

Our results showed that AARs of ICM were associated with a history of adverse reactions to ICM, history of various allergies, age, ICM type, CT examination type, cardiac insufficiency and hyperlipidemia. The elevated IgE might be potentially associated with acute allergic-like reactions. The EOS values and blood glucose values were not associated with AARs. This study advances the understanding of AARs risk by integrating clinical and biomarker data.

### 4.1 Risk factors of AARs

#### 4.1.1 A history of adverse reactions to ICM, history of various allergies, age, and cardiac insufficiency

Reportedly, a history of adverse reactions to ICM, history of various allergies, age, and cardiac insufficiency are associated with AARs (17–20), which is consistent with our findings.

#### 4.1.2 Hyperlipidemia

Although to the best of our knowledge, no studies have directly reported the relationship between hyperlipidemia and AARs, hyperlipidemia is reportedly a risk factor of Kounis syndrome, which is a coronary allergic-like disorder induced by exposure to drugs (e.g., antibiotics, anesthetics, and ICM), food, environment, and other triggers (e.g., insect bites and stings and poison ivy) (21), the three main variants of Kounis syndrome include vasospastic allergic angina, allergic myocardial infarction, and stent thrombosis with occluding thrombus infiltrated by EOS and mast cells (22), indicating the indirect relationship between hyperlipidemia and AARs to ICM, consistent with the results of this study. Hyperlipidemia, a potential preliminary novel risk factor, may exacerbate vascular allergic-like via mechanisms linked to Kounis syndrome. Patients with hyperlipidemia exhibit increased blood viscosity and slow blood flow. Notably, herein, the patients with hyperlipidemia were 3.134 times at a risk of AARs than those without hyperlipidemia.

Conducting our study in a cardiovascular specialty hospital introduces potential confounding from high rates of cardiovascular medication use (such as statins and beta-blockers). The high prevalence of hyperlipidemia is intrinsically linked to the underlying indications for contrast-enhanced CT (e.g., coronary heart disease, cerebrovascular disease, and renal complications). Therefore, the observed association between hyperlipidemia and AARs should be interpreted with caution, as it may be confounded by these factors or the underlying disease indications. Future research employing stratified analysis or matched comparisons is warranted to explore how medication bias and underlying disease profiles may influence these results.

#### 4.1.3 CT examination and ICM type

The results of this trial showed that the CCTA examination (vs. non-CCTA) and ICM type of iopromide (vs. iodixanol) were associated with the AARs of ICM. This might be related to the fact that the CCTA examinations were mainly accompanied by patients of cardiovascular diseases, which were considered to be at high risk of AARs to ICM (3). Some authors reported that the different types of contrast-enhanced CT scans were related to AARs (15), and iopromide was more likely to cause AARs (8) which were consistent with our results.

### 4.2 Risk factors of acute allergic-like reactions

#### 4.2.1 IgE

The relationship between IgE and AARs to ICM remains to be established. Some studies have suggested that IgE is associated with AARs to ICM (6, 8–10, 23, 24) and vice versa (25). To confirm this, further investigation of the relationship between IgE and acute allergic-like reactions is warranted.

The etiological mechanism underlying most allergic-like reactions to ICM remains unelucidated. It is generally accepted that most allergic-like reactions are not associated with high IgE levels and are therefore unlikely to be typical IgE-mediated allergic reactions (26). However, there is growing evidence (27) demonstrating that certain severe allergic-like reactions are IgE-mediated and positive for the skin test, whereas mild-to-moderate allergic-like reactions are mostly non-allergic. Furthermore, skin testing has been proved to be able to differentiate between allergic (i.e., IgE-mediated) and non-allergic reactions (28).

The mechanism underlying AARs to ICM is considered multifactorial. It is generally accepted that IgE is associated with type I acute immunologic response, a type of acute allergic reaction. Therefore, IgE is considered to be associated with acute allergic-like reactions to some extent. Elevated IgE supports IgE-mediated pathways in severe reactions, aligning with recent evidence (29). This study suggested that the baseline IgE levels of the patients were potentially associated with acute allergic-like reactions and might serve as a candidate predictor, consistent with the findings of previous studies. However, it is crucial to note that total serum IgE is inherently nonspecific and does not reflect a patient's sensitization status to ICM. Unlike allergen-specific IgE, total IgE levels provide only a general assessment and are significantly influenced by various factors, including atopic background, parasitic infections, and smoking habits. Consequently, in the absence of specific sensitization data—such as serum-specific IgE levels or skin test results—a total IgE cutoff value (e.g., 158.1 IU/mL) has limited utility as a standalone screening tool for individual patients. Clinical interpretation must account for these potential confounding variables. Further research incorporating specific sensitization testing is warranted to better elucidate the causal link between IgE-mediated mechanisms and AARs to ICM.

While IgE-mediated mechanisms are the most well-established and studied pathways in ICM allergic-like reactions, it is important to acknowledge that other mechanisms, such as non-IgE-mediated pathways, complement-mediated pathways, cytokine-mediated pathways, and direct toxicity, may also contribute to the development of AARs. Further research is needed to better understand the relative contributions of these different mechanisms and to develop more effective strategies for the prevention and management of ICM allergic-like reactions.

#### 4.2.2 EOS

Reportedly, the baseline EOS level is high in patients with allergies, such as asthma (12, 13), consistent with the findings of this study, our findings indicated that EOS level is somewhat related to acute allergic-like reactions; however, it is not an independent predictor for the same. Nevertheless, this still requires further investigation.

#### 4.2.3 Gout

Although our multivariate analysis identified gout as a potential independent predictor of acute allergic-like reactions to ICM (OR = 29.48), this finding must be interpreted with significant caution. The wide confidence interval (2.98–291.82) is likely an artifact of data sparsity, given the low frequency of gout within the allergic-like reaction group. Consequently, the clinical mechanism linking gout to acute ICM allergic-like reaction remains speculative. Future studies with larger, more diverse cohorts are essential to validate this association and determine whether it represents a true clinical risk factor or an incidental observation influenced by small-sample bias.

### 4.3 Other risk factors

#### 4.3.1 Blood glucose

Furthermore, previous studies have reported that patients with debility or medical instability exhibit increased risk of allergic-like reactions (3). In addition, before contrast-enhanced CT, some patients need to fast for 4–6 h and some others for longer periods, such as those with pancreatitis, whose relationship between allergic-like reactions and blood glucose levels was evaluated via our test. Our findings indicate that there was no significant correlation between immediate blood glucose levels and acute allergic-like reactions to ICM. Reports in the literature supporting this specific observation are scarce. Some author (15) has reported that fasting is not an influence factor for the occurrence of AARs, but there is no mention of blood glucose.

#### 4.3.2 Others

The role of other risk factors, such as the ICM injection flow rate and other factors, determined herein were consistent with or slightly different from those reported in previous studies (23, 30). Notably, physiologic reactions (e.g., dizziness) likely reflect autonomic responses rather than immunopathology, warranting distinct clinical management. While our study focused on the risk factors for overall AARs, we acknowledge that grouping these distinct reaction types may obscure specific associations. Future studies with larger sample sizes and multi-center designs will allow for the separate analysis of physiologic and allergic-like reactions. Such stratification will be essential to identify more granular and clinically meaningful predictors that distinguish true allergic-like reactions from transient vasovagal or physiologic side effects.

### 4.4 Explanation of the research methods and research content of the trial

ROC analysis of the diagnostic value of the independent risk factors of AARs to ICM and those of allergic-like and physiologic reactions were not performed for the 1,462 patients included. For the patients whose blood glucose levels (1,077), IgE levels (770), and EOS levels (770) were measured, allergic-like reactions were analyzed as a potential risk factor and independent risk factor and their ROC curve was determined to evaluate their diagnostic value without corresponding analysis of AARs or physiologic reactions. Based on the growing evidence regarding the association of IgE with allergic-like reactions (19), although the incidence of high IgE levels was only 4% in patients with allergic-like symptoms (9), IgE was analyzed in this study. We did not conduct an in-depth analysis of the other risk factors, apart from the history of adverse reactions to ICM, owing to their uncertainty (19), including hyperlipidemia, which was mentioned in this study. Therefore, follow-up and further studies are warranted.

### 4.5 Limitations

This study has several limitations that warrant consideration.

(1) ICM diversity: We utilized two widely-used ICM types (iopromide and iodixanol), representing hypotonic and isotonic categories. While our sample size precluded a direct comparative analysis between them, their inclusion reflects current clinical practice. Future studies with a broader range of ICM and larger cohorts will further refine these findings.
(2) Study design and selection bias: Conducted at a single center with convenience sampling based on informed consent, the study acknowledges potential volunteer bias in biomarker testing. We compared the baseline characteristics between the included participants (with biomarker data) and the excluded participants (without biomarker data) (Supplementary Appendix 3). Future multi-center studies with diverse cohorts are planned to mitigate selection bias and enhance generalizability.
(3) Small valid sample size: Following the completion of experimental data collection and analysis, a *post-hoc* power analysis was conducted. The corresponding specific results are provided in Supplementary Appendix 2. We acknowledge that the limited number of effective samples for rare events may affect the robustness of the results. To mitigate this, we have employed methods specifically designed to handle rare events and reduce small-sample bias, such as Firth regression. The current sample represents the best feasible exploratory analysis based on available data, and that future multi-center studies with larger sample sizes are required for validation.
(4) Serum-specific IgE: While Serum-specific IgE measurements were not included, their potential to offer further insights into allergic status and strengthen causal evidence is acknowledged. Future research on Serum-specific IgE may provide additional valuable information.
(5) Model validation: Internal validation via bootstrapping (1,000 repetitions) was performed. However, external validation is crucial for confirming generalizability. A planned multi-center study with an expanded sample size aims to externally validate these models for broader clinical applicability.
(6) Confounding by indication and co-medications: The patient cohort from a specialized cardiovascular center inherently involves comorbidities (e.g., hyperlipidemia, cardiac insufficiency) and concomitant medications (e.g., beta-blockers) that may influence clinical outcomes. Future studies incorporating systematic pharmacological tracking will be essential to further clarify these interactions.

Despite these limitations, our study provides valuable exploratory data on potential risk factors for ICM-induced AARs.

## 5 Conclusion

This study identifies cardiac insufficiency, age, ICM type, CT examination type, a history of adverse reactions to ICM, and history of various allergies as critical risk factors for AARs. Our findings also hint at potential exploratory associations: between hyperlipidemia and AARs, and between IgE levels, ICM type, and gout, with allergic-like reactions. Given the non-specific nature of total IgE and the complex mechanisms involved, these specific associations should be strictly interpreted as exploratory hypothesis requiring confirmation through large-scale, multi-center studies with external validation. Before these findings can be considered for integration into pre-procedural screening protocols, further rigorous research is essential to establish their clinical utility.

## Author's note

The scientific guarantor of this publication is Ruigang Xie and Yinghui Ge.

## Data Availability

The original contributions presented in the study are included in the article/Supplementary Material, further inquiries can be directed to the corresponding author.
