# Supplementary material for: Prospective evaluation of risk factors for acute adverse reactions to iodinated contrast media in contrast-enhanced CT
Source: Front Med (Lausanne). 2026 Oct 7;13:1870320. doi: 10.3389/fmed.2026.1870320 (PMC13693528; doi:10.3389/fmed.2026.1870320)
Supplement: Supplementary file 1 [file Table1.docx]

**Appendix**

Appendix 1:

A Priori Sample Size Justification

we performed sample size estimation using Riley method, comprehensively calculating the required sample size from multiple dimensions (such as model overfitting degree, predictive accuracy). We implemented this using the pmsampsize package in R with the following parameters:

p: Number of candidate predictive factors 7;

R2: Expected R² for AARs (usually set between 0.1–0.2; set to 0.15 in this study);

p0: Incidence of the outcome event, estimated to be approximately 0.04 based on our institution’s clinical practice, expert consensus and the the literature (ranging from 0.2% to 6.0%);

Shrinkage: The target shrinkage factor was set to 0.9, allowing for 10% overfitting shrinkage.

The results showed that the sample size should be at least 1397, with at least 60 positive outcome events.

Appendix 2:

Post-hoc Power Analysis

We conducted a post-hoc power analysis based on the observed effect sizes.

Since there is no specific power calculation package for Firth regression, we utilized the WebPower package in R. This package is widely used, well-documented, supports multivariable models, and provides stable parameter estimates for logistic regression power analysis..

Calculation method: We used the observed odds ratios (OR) in the multi-factor Firth regression model. For the strongest predictive factor “history of contrast reaction,” the observed OR was 29.02. Using the sample size calculation method for logistic regression described by Hsieh et al., with a total sample size of N=1462 and an event occurrence rate of 4.2%, the posterior power to detect such a large effect size exceeded 99%.

However, acknowledging that this variable may exhibit quasi-complete separation, we conservatively selected hyperlipidemia (OR = 3.27) for the power analysis, which yielded a post-hoc power of 64.37%. Although this is below the conventional threshold of 80%, it is important to note that the outcome events in this study are rare. The OR values were derived from Firth regression, whereas the Power estimation relied on standard Logistic regression’s Power test method. Standard maximum likelihood estimation would likely fail or produce biased estimates.

Appendix 3:

The comparative analysis, detailed in the following Table (Baseline characteristics of patients with and without IgE testing), revealed a statistically significant difference in contrast type between the two groups (p < 0.001). Specifically, the included group had a higher proportion of patients who received contrast type of Iodixanol, while the excluded group had a higher proportion of patients who received contrast type of Iopromide.

This finding suggests that the selection of patients for biomarker testing was not completely random and may have introduced a degree of selection bias. However, we would like to clarify that the primary objective of our biomarker analysis was to explore the association between IgE, EOS, and glucose levels and acute adverse reactions within the subgroup of patients who underwent these specific tests. Our main predictive model for AARs, which is the primary focus of the manuscript, was developed using the entire cohort (n=1462) and did not rely on these biomarkers. Additionally, although there were statistically significant differences in contrast type, these differences were of limited clinical significance.

**Table** Baseline characteristics of patients with and without IgE testing

| **Variables** | **Total (n = 1462)** | **0 (n = 692)** | **1 (n = 770)** | **Statistic** | ***P*** |
| --- | --- | --- | --- | --- | --- |
|  |  |  |  |  |  |
| Age, M (Q₁, Q₃) | 60.00 (51.00, 68.00) | 60.00 (51.00, 68.00) | 60.00 (51.00, 69.00) | Z=-1.47 | 0.141 |
| Height, M (Q₁, Q₃) | 165.05 (159.00, 171.00) | 166.00 (159.23, 171.50) | 165.00 (159.00, 170.38) | Z=-1.35 | 0.176 |
| Weight, M (Q₁, Q₃) | 70.00 (60.20, 78.00) | 70.00 (61.00, 79.00) | 69.20 (60.00, 77.00) | Z=-1.55 | 0.121 |
| BMI, M (Q₁, Q₃) | 25.42 (23.18, 27.73) | 25.62 (23.47, 27.91) | 25.34 (22.95, 27.64) | Z=-1.21 | 0.225 |
| SBP, M (Q₁, Q₃) | 132.00 (120.00, 147.00) | 134.00 (121.00, 146.00) | 131.50 (120.00, 147.00) | Z=-1.25 | 0.21 |
| DBP, M (Q₁, Q₃) | 84.00 (76.00, 93.00) | 84.00 (78.00, 93.00) | 84.00 (75.00, 93.00) | Z=-1.87 | 0.062 |
| Contrast volume, M (Q₁, Q₃) | 60.00 (60.00, 80.00) | 65.00 (60.00, 80.00) | 60.00 (60.00, 80.00) | Z=-0.10 | 0.921 |
| Njectionrate, M (Q₁, Q₃) | 4.50 (4.00, 4.50) | 4.50 (4.00, 4.50) | 4.50 (4.00, 4.50) | Z=-1.03 | 0.302 |
| Creatinine, M (Q₁, Q₃) | 69.00 (58.00, 81.00) | 69.00 (57.00, 81.00) | 69.00 (58.00, 80.00) | Z=-0.33 | 0.741 |
| Sex, n(%) |  |  |  | χ²=0.02 | 0.897 |
| 1 | 911 (62.31) | 430 (62.14) | 481 (62.47) |  |  |
| 2 | 551 (37.69) | 262 (37.86) | 289 (37.53) |  |  |
| Contrast type, n(%) |  |  |  | χ²=67.13 | **<.001** |
| 1 | 872 (59.64) | 336 (48.55) | 536 (69.61) |  |  |
| 2 | 590 (40.36) | 356 (51.45) | 234 (30.39) |  |  |
| CCTA, n(%) |  |  |  | χ²=0.75 | 0.388 |
| 1 | 596 (40.77) | 274 (39.60) | 322 (41.82) |  |  |
| 2 | 866 (59.23) | 418 (60.40) | 448 (58.18) |  |  |
| Hypertension, n(%) |  |  |  | χ²=0.58 | 0.447 |
| 0 | 740 (50.62) | 343 (49.57) | 397 (51.56) |  |  |
| 1 | 722 (49.38) | 349 (50.43) | 373 (48.44) |  |  |
| CAD, n(%) |  |  |  | χ²=0.71 | 0.4 |
| 0 | 826 (56.50) | 383 (55.35) | 443 (57.53) |  |  |
| 1 | 636 (43.50) | 309 (44.65) | 327 (42.47) |  |  |
| Hyperlipidemia, n(%) |  |  |  | χ²=1.65 | 0.2 |
| 0 | 1331 (91.04) | 623 (90.03) | 708 (91.95) |  |  |
| 1 | 131 (8.96) | 69 (9.97) | 62 (8.05) |  |  |
| Heartfailure, n(%) |  |  |  | χ²=3.22 | 0.073 |
| 0 | 1123 (76.81) | 546 (78.90) | 577 (74.94) |  |  |
| 1 | 339 (23.19) | 146 (21.10) | 193 (25.06) |  |  |
| Diabetes, n(%) |  |  |  | χ²=0.71 | 0.399 |
| 0 | 1136 (77.70) | 531 (76.73) | 605 (78.57) |  |  |
| 1 | 326 (22.30) | 161 (23.27) | 165 (21.43) |  |  |
| Nephropathy, n(%) |  |  |  | χ²=1.18 | 0.278 |
| 0 | 1403 (95.96) | 660 (95.38) | 743 (96.49) |  |  |
| 1 | 59 (4.04) | 32 (4.62) | 27 (3.51) |  |  |
| Thyroiddisorders, n(%) |  |  |  | χ²=0.11 | 0.737 |
| 0 | 1231 (84.20) | 585 (84.54) | 646 (83.90) |  |  |
| 1 | 231 (15.80) | 107 (15.46) | 124 (16.10) |  |  |
| Gout, n(%) |  |  |  | χ²=0.00 | 1 |
| 0 | 1453 (99.38) | 688 (99.42) | 765 (99.35) |  |  |
| 1 | 9 (0.62) | 4 (0.58) | 5 (0.65) |  |  |
| Allergic diseases, n(%) |  |  |  | χ²=1.82 | 0.177 |
| 0 | 1419 (97.06) | 676 (97.69) | 743 (96.49) |  |  |
| 1 | 43 (2.94) | 16 (2.31) | 27 (3.51) |  |  |
| Priorcontrast reaction, n(%) |  |  |  | χ²=1.03 | 0.309 |
| 0 | 1457 (99.66) | 688 (99.42) | 769 (99.87) |  |  |
| 1 | 5 (0.34) | 4 (0.58) | 1 (0.13) |  |  |
| Allergyhistory, n(%) |  |  |  | χ²=0.33 | 0.568 |
| 0 | 1356 (92.75) | 639 (92.34) | 717 (93.12) |  |  |
| 1 | 106 (7.25) | 53 (7.66) | 53 (6.88) |  |  |
| Z: Mann-Whitney test, χ²: Chi-square test | | | | | |
| M: Median, Q₁: 1st Quartile, Q₃: 3st Quartile | | | | | |

Appendix 4:

The multivariable Firth regression backward stepwise selection analysis results

| Characteristic | β | se | *P* | OR | 95% *CI* |
| --- | --- | --- | --- | --- | --- |
| Intercept | -2.47 | 0.61 | <0.001 | 0.08 | (0.03, 0.28) |
| Age(y) | -0.02 | 0.01 | 0.048 | 0.98 | (0.96, 0.999) |
| Cardiac insufficiency | 0.68 | 0.29 | 0.026 | 1.98 | (1.12, 3.52) |
| Contrast media type | 0.8 | 0.27 | 0.028 | 2.22 | (1.32, 3.74) |
| CT examination type CCTA vs Non-CCTA | -0.71 | 0.27 | 0.010 | 0.49 | (0.29, 0.84) |
| Hyperlipidemia | 1.14 | 0.32 | <0.001 | 3.13 | (1.69, 5.82) |
| History of adverse reaction to ICM | 3.57 | 0.87 | <0.001 | 35.41 | (6.41, 195.7) |
| History of various allergies | 1.20 | 0.36 | 0.002 | 3.33 | (1.65, 6.69) |

Appendix 5:
Sensitivity analysis

| Characteristic | β | se | *P* | OR | 95% CI |
| --- | --- | --- | --- | --- | --- |
| Intercept | -2.47 | 0.6 | <0.001 | 0.08 | (0.03, 0.27) |
| Age(y) | -0.02 | 0.01 | 0.064 | 0.98 | (0.96, 1) |
| Cardiac insufficiency | 0.68 | 0.29 | 0.026 | 1.97 | (1.12, 3.47) |
| Contrast media type | 0.78 | 0.26 | 0.030 | 2.19 | (1.31, 3.65) |
| CT examination type CCTA vs Non-CCTA | -0.75 | 0.27 | 0.005 | 0.47 | (0.28, 0.8) |
| Hyperlipidemia | 1.08 | 0.31 | 0.002 | 2.93 | (1.59, 5.42) |
| History of various allergies | 1.23 | 0.35 | 0.002 | 3.42 | (1.72, 6.7958) |
