# Supplementary material for: Prospective evaluation of risk factors for acute adverse reactions to iodinated contrast media in contrast-enhanced CT
Source: Front Med (Lausanne). 2026 Oct 7;13:1870320. doi: 10.3389/fmed.2026.1870320 (PMC13693528; doi:10.3389/fmed.2026.1870320)
Supplement: Supplementary file 2 [file Table2.docx]

**Attachment 1**

**The questionnaire about contrast-enhanced CT**

(Note: this questionnaire is need to be filled in after 60 minutes when contrast-enhanced CT completed)

Name: Medical record number: Telephone number:

Do you have any symptoms such as the following before performing examination? (Please tick in the relevant box)

| 1 | Acceleration of heartbeat, sweating, frequency/urgency  of urination, epigastric discomfort, etc.? | Yes □ | No □ |
| --- | --- | --- | --- |

Do you have new symptoms such as the following after performing examination? (Please tick in the relevant box)

| 1 | Limited urticaria/pruritus? | Yes □ | No □ |
| --- | --- | --- | --- |
| 2 | Limited erythema/cutaneous edema? | Yes □ | No □ |
| 3 | Limited itchy throat? | Yes □ | No □ |
| 4 | Limited nausea/vomiting? | Yes □ | No □ |
| 5 | Limited transient flushing/warmth/chills? | Yes □ | No □ |
| 6 | Altered taste? (Metallic taste, etc.) | Yes □ | No □ |
| 7 | Lip swelling/edema? | Yes □ | No □ |
| 8 | Dizziness/headache? | Yes □ | No □ |
| 9 | Chest tightness/dyspnea? | Yes □ | No □ |

**Attachment 2** IgE assay performance: Detailed data on assay precision and reproducibility.

**Attachment 3** IgE internal quality control: Laboratory Information System (LIS) records from November 2020.

**Attachment 4** EOS assay performance: Detailed data on assay precision and reproducibility.

**Attachment 5** EOS internal quality control: Laboratory Information System (LIS) records from November 2020.
